# Patients with HFpEF and HFrEF have different clinical characteristics but similar prognosis: a retrospective cohort study

**DOI:** 10.1186/s12872-016-0418-9

**Published:** 2016-11-21

**Authors:** Tamrat Befekadu Abebe, Eyob Alemayehu Gebreyohannes, Yonas Getaye Tefera, Tadesse Melaku Abegaz

**Affiliations:** Department of Clinical Pharmacy, School of Pharmacy, College of Medicine and Health Science, University of Gondar, Gondar, Ethiopia

**Keywords:** Heart failure, Ejection fraction, Clinical characteristics, Survival, Ethiopia

## Abstract

**Background:**

Globally, heart failure (HF) has been recognized as one of the major cardiovascular disorder with high morbidity, mortality and considerable social impact. In Sub Saharan African countries, HF has turned out as a leading form of cardiovascular diseases, and has considerable socioeconomic impact. However, there are differences in clinical characteristics and survival status among patients with preserved (HFpEF) and reduced (HFrEF) ejection fraction. The aim of this study is to outline the clinical characteristics and medication profile, assess the survival status and prognostic factors of Ethiopian HF patients with HFrEF and HFpEF.

**Methods:**

A retrospective cohort study was carried out and we employed medical records of patient’s, admitted as a result of HF to the University of Gondar Referral Hospital in the period between December 02, 2010 and December 01, 2015 due to HF. Kaplan Meier curve was used to analyze the survival status and log rank test was used to compare the curves. Cox regression was used to analyze independent predictors of mortality in all HF patients.

**Results:**

Of the 850 patients who were admitted due to HF, 311 patients met the inclusion criteria. Majority of the patients had HFpEF (52.73%) and tend to be women (76.22%). They predominantly had etiologies of valvular and hypertensive heart diseases, and took calcium channel blockers and anticoagulants. Conversely, patients with HFrEF had etiologies of ischemic heart disease and dilated cardiomyopathy and were prescribed angiotensine converting inhibitors (ACEI) and beta blockers. Kaplan Meier curves and Log rank test (*p* = 0.807) showed that there was no statistically significant difference in the mortality difference among patients with HFpEF and HFrEF. On the other hand, Cox regression analysis showed advanced age, lower sodium level, higher creatinine level and absence of medications like ACEI, spironolactone and statins independently predicted mortality in all HF patients.

**Conclusions:**

Different clinical characteristics were found in both groups of HF patients. There was no difference in survival outcome between patients with HFrEF and HFpEF.

## Background

Heart failure (HF) is one of the major cardiovascular disorder with high morbidity and mortality and rising costs, which account for 1–2% of the annual health budget of most developed nations [1, 2]. HF primarily involves (affects) people with advanced age, and incidence and prevalence increases progressively in those age > 60 years [3]. The most frequent prevalence estimate for the adult population at large is 2% and more than 10% particularly in those aged 75 years and older [2, 4–6]. In Sub Saharan African countries (SSA), HF has turned out as a leading form of cardiovascular disease, and has considerable socioeconomic impact owing to its high prevalence, mortality and impact on young generations [7]. Unlike developed nations, the most prevailing causes of HF in Africans remains largely non ischemic [7]. Two third of HF cases in SSA are mainly secondary to Hypertension (HTN), cardiomyopathy and rheumatic heart disease [7, 8]. In contrast, in industrialized nations, coronary artery disease, either alone or in combination with HTN, is the prominent cause of HF [9, 10].

Left ventricular dysfunction (LVD) is categorized into systolic and diastolic dysfunction. A LVEF above 50% and below 40 are considered reserved and reduced ejection fractions, respectively, leaving an intermediary range of 40 to 50% [11]. Irregularity in the filling properties or capacity of the left ventricle in patients with LVEF > 50 is used to classify diastolic dysfunction [11, 12]. HF is usually considered typical when presenting in patients with dilated hearts and systolic dysfunction (ejection fraction (EF) < 40%). As a result, the most therapeutic evidence, including effective pharmacological and device therapies that have led to impressive improvements in survival is in this group of patients with HFrEF. By contrast, most clinical trials excluded patients with HF with preserved ejection fraction (HFpEF > 50%) [11, 12].

In the past three decades, prevalence of HF is on the rise.[3, 13, 14]. Demographically, prevalence of HF has certain difference between patients with systolic dysfunction (SDf) and diastolic dysfunction (DDf). Most patients with DDf are usually older age and female, and have left ventricular hypertrophy and arterial hypertension [15–18]. Moreover, there are disparities on the prognosis of patients with SDf and DDf as some researches demonstrated that morbidity and mortality is worse in the earlier group while other studies emphasize that there is no convincing discrepancy in prognosis between these groups [13, 19–22]. To the best of our knowledge, unlike western countries, we did not found sufficient studies on the clinical characteristics of patients with HF with reduced ejection fraction (HFrEF) and HFpEF in SSA countries. Hence, we aimed to evaluate HF patient’s clinical characteristics and outcome, and medication profile based on patients recorded EF during admission to Gondar University Referral Hospital (GURH), Ethiopia.

## Methods

### Study population

We retrospectively assessed medical transcript of patients who had been admitted with a diagnosis of HF in Internal Medicine ward of GURH in the period between December 02, 2010 and December 01, 2015. Patients who had been diagnosed HF, are 18 years of age or older, and with presence of two or more of the adapted Framingham criteria or presence of one major criteria and two minor criteria has been included in the study. Major criteria include: presence of sign and symptoms of raised jugular venous pressure, paroxysmal nocturnal dyspnea, clinical cardiomegaly confirmed by chest X- ray, basal crepitation, third heart sound, pulmonary edema confirmed by chest X- Ray, hepatojugular reflux and acute pulmonary edema. Minor criteria include: heart rate >120 beats per minute, orthopnea, exertional dyspnea, nocturnal cough, hepatomegaly, pleural effusion, and peripheral edema. Exclusion criteria were patients who had concomitant infection in addition to HF, patients who did not have echocardiography record, patients who were New York Heart Association (NYHA) Class I and class II during admission and patients who did not have laboratory analysis records on complete blood count, electrolytes and creatinine level. The internal medicine ward received around 850 patients during the study period and 539 were excluded from the study based on the exclusion criteria.

Patients were grouped based on their LVEF record into two: patients with HFpEF (LVEF ≥ 50%) and patients with HFrEF (LVEF < 50%). Ejection fraction of the participants were measured using echocardiogram during their first admission to the internal medicine ward by a radiologist. Survival status of the patients were recorded based on their vital status record on last hospital discharge from internal medicine ward or the last time patient’s came for routine checkup or to refilled their medication. Patient’s data was collected by two clinical pharmacists (co – authors) during the study period.

Hypertension was determined as blood pressure 140/90 mmHg or more, anemia as hemoglobin level < 13 g/dl for men and <12 g/dl for women.

The etiology of HF was determined based on the following algorism: Ischemic, when the patient was diagnosed with Ischemic heart disease (IHD); Valvular, when there was moderate valulopathy with no IHD; hypertensive, when there was previous history of hypertension but no evidence of additional cardiovascular disease; dilated cardiomyopathy (DCMP), when there was no other known cardiac cause and had LVEF < 50%; and *Cor pulmonale*, when right sided heart failure without left ventricular dysfunction is present.

### Statistics

Statistical analysis was conducted for both quantitative and qualitative variables. Group variables were indicated in percentage and Chi square test was used. A probability of type I error <5% was considered significant. Continuous variables were revealed in mean ± SD and independent t – test was used with a probability of type I error < 5% considered significant. Kaplan – Meier survival curves were designed and analyzed with the Mantel log rank test to measure the significance. Univariate Cox regression analysis was used to determine the co-factors with momentous outcome on all-cause mortality. Multivariate Cox regression analysis was implemented to determine the independent foreboding factors for all-cause death. All data were analyzed with SPSS version 20.0 (SPSS Inc. Chicago, IL) software for windows.

## Results

Of 850 patients who were admitted to GURH due to HF, in the period between December 02, 2010 and December 01, 2015, 311 patients met the inclusion criteria. From the study group, 164 patients had HFpEF (EF ≥ 50%) and the remaining participants (147) had HFrEF (EF < 50%). Table 1 shows baseline clinical characteristics of the two groups. The mean age of the participants were 53.58 (±16.902) years with no significant divergence among the groups (54.20 (±16.587) Vs.52.88 (±17.246), *P* = 0.496), and women accounted for the majority of patients with LVEF ≥ 50 (76.22% vs. 62.59%, *P* = 0.009). Analysis of cardiovascular risk factors such as hypertension and diabetes, other co morbidities like anemia, atrial fibrillation, and NYHA functional class revealed no compelling variance among the groups. However, heart failure etiology clearly distinguished between the HFrEF and HFpEF groups. In the earlier groups Ischemic heart disease (IHD) (21.09% vs. 10.98%, *P* = 0.015) and DCMP (21.77% vs. 4.27%, *P* = <0.001) were more prevalent; whereas, VHD (34.01% vs. 45.96%, *P =* 0.020) and HHD (11.56% vs. 20.12%, *P* = 0.040) were more prevalent in the later groups. There were also significant differences between patients with HFpEF and HFrEF in their heart rate (95.60 (±21.177) Vs.89.93 (±16.287), *P* = 0.009).

**Table 1:** Clinical characteristics of Heart Failure patients based on Preserved of reduced ejection fraction

Variable	Total patients (*n* = 311)	LVEF ≥ 50% (*n* = 164)	LVEF < 50% (*n* = 147)	*P* value
Women, n (%)	217 (69.77)	125 (76.22)	92 (62.59)	0.009
Age, Years (mean ± SD)	53.58 (±16.902)	54.20 (±16.587)	52.88 (±17.246)	0.496
Anemia, n (%)	118 (37.94)	65 (39.63)	53 (36.05)	0.516
AF, n (%)	79 (25.41)	47 (28.66)	32 (21.77)	0.163
DM, n (%)	4 (1.29)	2 (1.22)	2 (1.36)	0.912
Hypertension, n (%)	97 (0.312)	56 (34.15)	41 (27.89)	0.235
Heart Rate ( mean ± SD)	92.88 (±19.166)	95.60 (±21.177)	89.93 (±16.287)	0.009
Systolic BP	122.75 (±24.231)	125.06 (±26.911)	120.18 (±20.651)	0.072
NYHA Functional Class				0.274
Class III, n (%)	82 (26.37)	39 (23.78)	43 (29.25)	
Class IV, n (%)	229 (73.63)	125 (76.22)	104 (70.75)	
Etiology				
IHD, n (%)	49 (15.76)	18 (10.98)	31 (21.09)	0.015
HHD, n (%)	50 (16.08)	33 (20.12)	17 (11.56)	0.040
VHD, n (%)	127 (40.84)	77 (46.95)	50 (34.01)	0.020
DCMP, n (%)	39 (12.54)	7 (4.27)	32 (21.77)	<0.001
Cor pulmonale, n (%)	14 (4.50)	9 (5.48)	5 (3.40)	0.376
Others, n (%)	32 (10.29)	20 (12.20)	12 (8.16)	0.243

*AF* atrial fibrillation, *BP* blood pressure, *DCMP* dilated cardiomyopathy *DM* diabetes mellitus, *HHD* hypertensive heart disease, *IHD* ischemic heart disease, *NYHA* New York Heart Association, *SD* standard deviation

### Medical treatment

Table 2 compares treatments at discharge in patients in the two groups. There were no significant difference in the prescription of diuretics, spironolactone, antiplatelet agents, digoxin and statins. Patients classified as having depressed LVEF were usually prescribed angiotensin converting enzyme inhibitor (ACEI) and beta blockers. On the other hand, anti-coagulants and calcium channel blockers were more frequently prescribed in patients with HFpEF.

**Table 2:** Medication profile of heart failure patients based on ejection fraction

Medication	LVEF ≥ 50 (n,164)	LVEF < 50% (n,147)	*P* value
ACEI n, %	34 (15.85)	80 (40.82)	<0.001
Beta Blocker n, %	45 (21.95)	73 (36.73)	<0.001
Diuretics n, %	146 (74.39)	136 (70.75)	0.290
Spironolactone n, %	114 (57.93)	95 (48.99)	0.359
Digoxin n, %	46 (23.17)	33 (17.69)	0.257
Statin n, %	24 (11.59)	30 (13.61)	0.180
Anticoagulants n, %	33 (17.07)	12 (14.70)	0.003
Antiplatelet agents n, %	33 (15.24)	39 (17.69)	0.181
CCB n, %	21 (9.76)	7 (4.76)	0.013

*ACEI* angiotensin converting enzyme inhibitor, *CCB* calcium channel blocker

### Results of laboratory analyses and echocardiograms

Higher hemoglobin levels were measured in patients with reduced ejection fraction than preserved ejection fraction (13.31 ± 3.76 vs. 12.47 ± 3.08, *P* = 0.031). Greater ventricular dimension were recorded in patients with HFrEF (57.38 ± 9.07 vs. 46.10 ± 8.64, P = < 0.001). The mean EF in patients with preserved and reduced EF were 62.57 ± 8.62 and 40.36 ± 6.81respectively (Table 3).

**Table 3:** Laboratory and echocardiography results of heart failure patients based on ejection fraction

Variable	LVEF ≥ 50 (n,164)	LVEF < 50% (n, 147)	*P* value
Hgb (Mean ± SD)	12.47 ± 3.08	13.31 ± 3.76	0.031
Cr. (Mean ± SD)	1.1086 ± 0.774	1.0690 ± 0.536	0.598
Sodium (Mean ± SD)	134.86 ± 5.44	135.31 ± 7.92	0.563
LVEDD in mm (Mean ± SD)	46.10 ± 8.64	57.38 ± 9.07	<0.001
LVEF% (mean ± SD)	62.57 ± 8.62	40.36 ± 6.81	<0.001

*Hgb* hemoglobin, *Cr* creatinine, *LVEDD* left ventricular end diastolic diameter, *LVEF* left ventricular ejection fraction, *SD* standard deviation

### Survival analysis

**Fig. 1:**
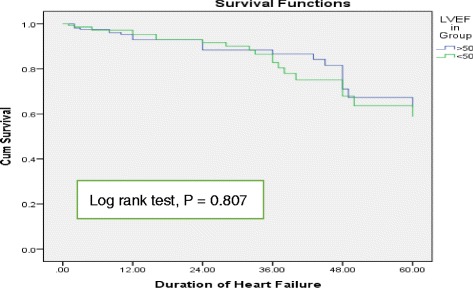
Kaplan Meier survival curves at 60 months for patients with preserved and reduced ejection fraction

The mean duration of follow up was 24.56 ± 17.79 months. Mortality were 14.02% in patients with HFpEF (23 patients) and 14.29% in patients with HFrEF (21 patients). Kaplan Meier survival curves (Fig. 1) shows there were no significant difference in survival status of patients with HFrEF and HFpEF (Log Rank test, *P* = 0.807). On the other hand, univariate Cox regression analysis showed that age, sodium level, creatinine level, IHD, Spironolactone, and digoxin were significantly associated with morality in patients with HF (Table 4). Multivariate Cox regression analysis, including the significant predictors of mortality identified on univariate Cox regression, showed that advanced age, low sodium level, high creatinine level, and absences of medications (spironolactone, ACEI and satin) significantly predicted morality (Table 4).

**Table 4:** Predictors of mortality to all causes of heart failure

Univariate analysis			Multivariate analysis	
Variables		Hazard ratio (95%CI)	Hazard ratio (95%CI)	*P* value
Gender	Male	0.984 (0.521–1.857)	1.132 (0.463–2.766)	0.785
Age, Years	Age, years	1.046 (1.025–1.068)	1.045 (1.016–1.076)	0.002
NYHA Class	Class IV	1.549 (0.719–3.337)	1.267 (0.527–3.046)	0.597
Anemia	Yes	1.483 (0.818–2.689)	0.708 (0.333–1.504)	0.369
Sodium, mEq/L		0.933 (0.896–0.972)	0.913 (0.851–0.980)	0.011
Creatinine, mg/dl		2.602 (2.052–3.300)	1.966 (1.369–2.824)	<0.001
AF	Yes	0.486 (0.225–1.051)	0.617 (0.171–2.224)	0.461
VHD	Yes	0.636 (0.342–1.185)	1.420 (0.413–4.886)	0.578
IHD	Yes	2.355 (1.182–4.691)	2.211 (0.397–12.331)	0.365
DCMP	Yes	0.898 (0.352–2.290)	1.068 (0.240–4.743)	0.931
HHD	Yes	1.036 (0.481–2.229)	1.272 (0.338–4.794)	0.722
LVEF		0.997 (0.975–1.020)	0.992 (0.958–1.027)	0.634
Diuritics	Yes	0.877 (0.269–2.858)	0.690 (0.180–2.650)	0.589
Spironolactone	Yes	0.388 (0.214–0.703)	0.343 (0.158–0.743)	0.007
ACEI	Yes	0.952 (0.510–1.778)	0.258 (0.098–0.680)	0.006
Beta Blocker	Yes	1.205 (0.659–2.204)	1.713 (0.766–3.832)	0.190
Digoxin	Yes	0.313 (0.132–0.746)	0.407 (0.095–1.736)	0.224
Antiplatelates	Yes	0.883 (0.435–1.793)	1.383 (0.352–5.436)	0.643
Anticoagulants	Yes	1.700 (0.817–3.541)	2.429 (0.845–6.978)	0.099
Statin	Yes	1.520 (0.768–3.010)	0.189 (0.037–0.972)	0.046
CCB	Yes	1.555 (0.720–3.359)	0.815 (0.287–2.317)	0.701

*ACEI* angiotensin converting enzyme inhibitor, *AF* atrial fibrillation, *CCB* calcium channel blocker, *DCMP* dilated cardiomyopathy, *HHD* hypertensive heart disease, *IHD* ischemic heart disease, *LVEF* left ventricular ejection fraction, *NYHA* New York Heart Association, *VHD* valvular heart disease

Factors included in multivariate Cox model include: Gender, Age, NYHA Class, presence of comorbidities such as anemia and AF, Common etiologies of HF such as VHD, IHD,HHD, and DCMP, Use of medications such as Diuretics, Spironolactone, ACEI, Beta Blocker, Digoxin, Antiplatelates, Anticoagulants, Statin and CCB, and laboratory values such as sodium and creatinine

## Discussion

The present research analyzed data from a retrospective observational cohort of HF patients to assess clinical characteristics, medication profile and determinants of mortality among HF patients with HFrEF and HFpEF who were admitted at GURH.

The prevalence of HFpEF is on the raise with period in developed nations [13, 23], varies depending on the age of the patient, race and the cutoff values for LVEF. In spite of that, there is a pervasive agreement that HFpEF is prominent in more than a third of all patients admitted with HF [19, 24–27]. In our study, the prevalence of HFpEF was 52.73% which was in alignment with other studies [28–30]. A recent study conducted by L. Martínez-Bra˜na et al. found that prevalence of HFpEF in Spain was 72.2% [20]. A study carried out in Japan by H. Kaneko et al. reported that prevalence of HFpEF was 72% [31]. In the Identification of Patients With Heart Failure and PREserved Systolic Function: an epidemiological regionalstudy (I PREFER) prevalence of HFpEF was about 65% [32]. The disparity between these findings and our study might be credited to sample size variation of the study population, age of the patients, race and cutoff values for LVEF during the study.

Patients with HFpEF are at advanced age, women and more often have hypertension compared to patients with HFrEF [20, 25, 27, 31]. In a systematic review reported by E. E.S. van Riet et al., HFpEF was more prevalent with age > 60 years and female gender [1]. In a meta-analysis study, compared with patients with HFrEF, those with HFpEF were older and more often women, and have a history of hypertension [10]. The differences in patients’ backgrounds in our study were almost indistinguishable to those of previous articles as patients with HFpEF in this study were older and more often female.

Regarding the etiology of HF, hypertensive heart disease (HHD) and VHD are more common in patients with HFpEF. Whereas, in patients with HFrEF, the most prevalent causes were IHD and DCMP [19, 20, 24, 26]. A meta-analysis conducted by R.N. Doughty et.al reported that, compared with the HFrEF patients, those with HFpEF more often had a history of Hypertension and less likely had IHD [10]. In I PREFER, Irbesartan clinical trial and the Effects of Candesartan for the Management of Patients With Chronic Heart Failure (CHARM) program, it were reported that HHD was the most important etiologic factor for the diagnosis of HFpEF and CAD is the leading cause for patients with HFrEF [32–34]. Similar finding were documented in our study, where IHD and DCMP were the leading etiologies for the prevalence of HFrEF; in contrast, VHD and HHD were responsible for the diagnosis of HFpEF. One of the interesting finding in our study was, the impact of IHD as an etiology of HF in patients with reduced ejection fraction. According to prior literatures, most of the etiologies in African population were non ischemic [7]. This disparity might indicate that the progressive change in the epidemiological pattern of the disease through time and the modification of individuals’ life style.

In terms of drug treatment, ACEI, Beta – Blockers and Digitalis are mostly prescribed to patients with HFrEF [19, 26, 31, 35–37]. However, CCB are usually indicated for patients with HFpEF [38]. This is further supported by 2016 ESC European heart failure guideline, as indicated amlodipine in HF patients with preserved ejection fraction and ventricular rhythm problem [39]. Unlike HF with HFrEF, patients with HFpEF did not benefit from the novel medications indicated to manage the disease and improve morbidity and mortality. Hence, there are few clinical trials on this group of patients and trials already conducted on ACEI/Angiotensin Receptor Blockers showed that there is no promising influence on reducing the mortality rate in patients with HFpEF [33, 40]. The CHARM Program study showed no significant effect on mortality, but showed significant benefit in preventing HF hospitalization [34]. One of the proposed predictions by Y. Juillière et al. was systemic hypertension is more frequently associated with preserved ejection fraction, resulting a higher rate of prescription of CCB. In contrast most of the etiologies in patients with HFrEF are usually IHD [38]. Similar findings were presented in our study, where most of patients with HFpEF took CCB and anticoagulants since hypertension and AF were more prevalent in this group of patients. However, ACEI and Beta blockers were more recommended in patients with HFrEF.

There is no clear cut variation in the long term clinical outcomes of patients with HFpEF and HFrEF. Studies conducted by Owan TE et al., F.P. Brouwers et al. and H. Kaneko et al. reported that patient with HFpEF had better prognosis than those with HFrEF [13, 31, 41]. Nonetheless, no significant variation in mortality between patients with preserved and reduced LVEF were reported by other studies [19, 20, 26]. In the current study, there was no substantial difference (Log rank test, *P* = 0.807) in the survival status of patients with preserved and reduced ejection fraction. However, patients with HFpEF had a relatively better survival status.

In the current study, multivariate cox regression analysis exhibited that the independent prognosticators of all causes of death in patients with HF were advanced age, low sodium level, high creatinine level, and absence of medications like statins, ACEI, and Spironolactone. Our findings were consistent with various studies; in a prospective study conducted by Macín SM, et al. [19] and Ojeda S, et al. [26] in Spain and a retrospective study in USA by Owan TE et al. [13] reported that advanced age, lower level of sodium, higher serum creatinine level as a predictor of unfavorable outcome in HF patients. In a retrospective study done by H. Kaneko et al. [31] found that absence of statins in patients with HFrEF medication prescription as a prognosticator of mortality. In a randomized control trial conducted by Pitt B et al. [42], it was reported that aldosterone blockers had a momentous importance in lowering morbidity and mortality by decreasing atrial natriuretic peptide concentrations. The study of left ventricular dysfunction (SOLVD) determined that addition of ACEI in HF treatment had significantly decreased mortality and morbidity in patients with HFrEF [43].

### Study limitation

Our study had several limitations. First, this study was carried out in a single center so the outcomes of the research cannot be generalized to all medical centers. Second, the sample size of the study population may not be sufficient enough to detect statistically significant differences. Third, the definition of HFrEF and HFpEF in the current study was based on LVEF, and it therefore remains not certain whether the study participants had objective documentation of diastolic dysfunction, as characterized by guiding principle for the diagnosis of HFpEF.

Despite these limitations, we believe that our study provides vital information on the clinical features and prognosis of patients with HF. Moreover, it will give a blue print for further clinical research in the area.

## Conclusions

In the current study, majority of the patients presented with HFpEF. As in other studies, women, hypertensive and VHD etiologies were more predominant in patients with HFpEF and takes CCB and anticoagulants. Patients with HFrEF had IHD and DCMP etiologies. They usually took Beta blockers and ACEI. There was no compelling disparity in mortality between patients with HFrEF and HFpEF. However, older age, lower sodium level, higher creatinine level and absence of medications like ACEI, spironolactone and statins independently predicted mortality in all HF patients. These outcomes indicated that further research should be conducted and several factors should be considered for preserved or reduced LVEF management.

## Abbreviations

ACEI: Angiotensin-converting enzyme inhibitor
AF: Atrial fibrillation
CAD: Coronary artery disease
CCB: Calcium Channel Blockers
CHARM: The Effects of Candesartan for the Management of Patients With Chronic Heart Failure
Cr: Creatinine
DCMP: Dilated cardiomyopathy
DDf: Diastolic dysfunction
DM: Diabetes mellitus
EF: Ejection fraction
GURH: Gondar University Referral Hospital
HF: Heart failure
HFpEF: Heart Failure with preserved Ejection Fraction
HFrEF: Heart Failure with reduced Ejection Fraction
Hgb: Hemoglobin
HHD: Hypertensive heart disease
HTN: Hypertension
I PREFER: Identification of Patients With Heart Failure and PREserved Systolic Function: an epidemiological regional study
IHD: Ischemic heart disease
LVD: Left ventricular dysfunction
LVEDD: Left ventricular end diastolic dimension
LVEF: Left ventricular ejection fraction
NYHA: New York Heart Association
SD: Standard deviation
SDF: Systolic dysfunction
SOLVED: The study of left ventricular dysfunction
SSA: Sub Saharan Africa
VHD: Valvular heart disease
